# Wet Grinder-Treated Okara Improved Both Mechanical Properties and Intermolecular Forces of Soybean Protein Isolate Gels

**DOI:** 10.3390/gels8100616

**Published:** 2022-09-27

**Authors:** Yuya Arai, Katsuyoshi Nishinari, Takao Nagano

**Affiliations:** 1 Department of Food Science, Faculty of Bioresources and Environmental Sciences, Ishikawa Prefectural University, 1-308, Suematsu, Nonoichi 921-8836, Japan; 2 Glyn O. Phillips Hydrocolloids Research Centre, School of Food and Biological Engineering, Hubei University of Technology, Wuhan 430068, China; 3 Department of Food and Human Health Sciences, Graduate School of Human Life Science, Osaka City University, 3-3-138 Sugimoto Sumiyoshi, Osaka 558-8585, Japan

**Keywords:** wet-type grinder, okara, soybean protein isolate, gel, intermolecular forces

## Abstract

The application of okara treated by a wet-type grinder (WG) is discussed in this paper. We examined the effect of WG-treated okara on the mechanical properties and intermolecular forces in soybean protein isolate (SPI) gels. SPI gels were prepared with varying amounts of WG-treated okara, and compression tests were performed. Protein solubility was also examined by homogenizing the gel in four different solutions (S1, 0.6 M sodium chloride (NaCl); S2, 0.6 M NaCl and 1.5 M urea; S3, 0.6 M NaCl and 8.0 M urea; and S4, 1.0 M sodium hydroxide). The gel with WG-treated okara had higher breaking stress but not breaking strain. In contrast, the protein solubility in S3 was lower than those of the gel without okara or with WG-untreated okara. A negative correlation (*R*^2^ = 0.86) was observed between breaking stress and protein solubility in S3. These results suggest that WG-treated okara enhanced the hydrophobic interactions of SPI gels because protein solubilization by S3 is caused by the differences in hydrophobic interactions.

## 1. Introduction

Okara is a soybean residue and rich in dietary fiber, which contains mainly insoluble forms such as cellulose and hemicellulose [1]. In contrast, soybean protein isolate (SPI) is often used to replace animal proteins in food production because of sustainability assurance [2].

Many studies have been conducted to improve the physicochemical properties of cellulose and okara [3,4,5]. These studies suggested the usefulness of decreasing the particle size of okara and cellulose to improve the properties of protein-based gel food [6,7,8,9,10,11,12]. Ultra-high-pressure homogenization (UHPH) was applied to soybean flour for tofu production. UHPH decreased the particle size of soybean flour, and the hardness of tofu prepared from UHPH-treated soybean flour was similar to that of the control tofu [6]. The effect of nano-sized and micro-sized okara on the gel properties of tofu and silver carp surimi has been investigated [7,8]. Nano-sized okara was well distributed in the gel matrices and provided a less gritty mouthfeel than that of micro-sized okara [7]. The breaking force and penetration distance of the surimi gels increased with the increase in concentration of nano-sized okara. Light microscopy images showed that nano-sized okara was well distributed in surimi gels, whereas micro-sized okara was not [8]. Additionally, the effects of regenerated cellulose, bacterial cellulose (BC) microfibrils, and cellulose nano-crystals (CNC) on the gel properties of whey protein gels were studied. The addition of regenerated cellulose to acid-induced whey protein gels increased their storage modulus *G*′ [9]. BC microfibrils also increased the storage modulus *G′* of whey protein gels [10]. The water-holding capacity and gel strength of whey protein gels increased with the increase in CNC concentration from 0% to 1.0% [11].

In our previous study, we used a wet-type grinder (WG) to improve the physicochemical properties of okara because WG is used to produce nano-cellulose [4,13,14]. We examined its effect on the mechanical properties and water-holding capacity of magnesium chloride-induced SPI gels. When the suspensions of okara were treated with a WG, the viscosity increased, and the dispersion performance improved with increasing WG passage. The breaking stress, strain, and water-holding capacity of SPI gels also increased with the addition of WG-treated okara [12]. These results demonstrate that WG-treated okara improves the gel properties of SPI. However, the mechanism of action remains unclear.

In this study, the effects of WG-treated okara on the mechanical properties and intermolecular forces of WG-treated okara–SPI gels were investigated to explore the mechanism by which WG-treated okara improves the gel properties of SPI. SPI gels were prepared with varying amounts of WG-treated okara, and compression measurements were performed. Protein solubility was examined by homogenizing the gels in four different solutions to understand the intermolecular forces required to form WG-treated okara–SPI gels.

## 2. Results and Discussion

### 2.1. Effect of WG-Treated Okara on the Mechanical Properties of SPI Gels

**Figure 1:**
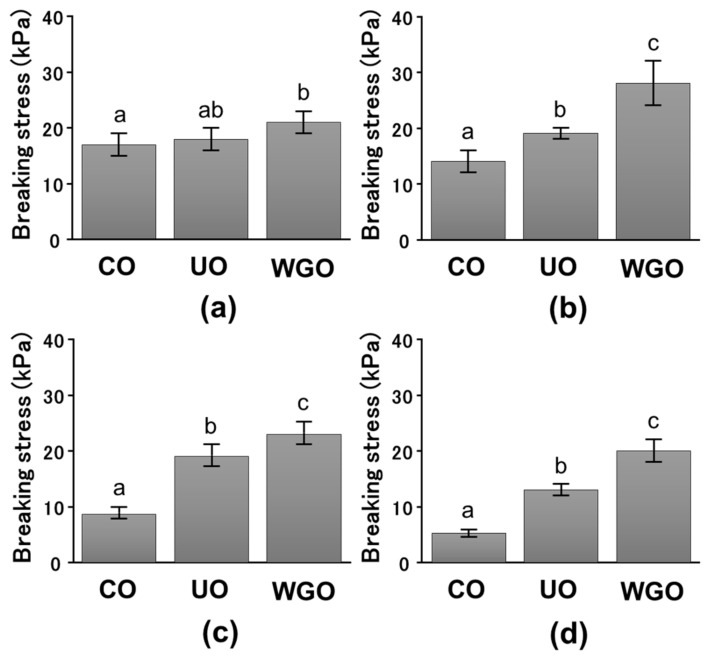
Breaking stress in okara–soybean protein isolate (SPI) gels. (**a**), 3% okara–SPI gels; CO, control SPI gel (15.52% SPI); UO, wet-type grinder (WG)-untreated okara–SPI gel (15.52% SPI and 0.48% WG-untreated okara); WGO, WG-treated okara–SPI gel (15.52% SPI and 0.48% WG-treated okara). (**b**), 5% okara–SPI gels; CO, control SPI gel (15.20% SPI); UO, WG-untreated okara–SPI gel (15.20% SPI and 0.80% WG-untreated okara); WGO, WG-treated okara–SPI gel (15.20% SPI and 0.80% WG-treated okara). (**c**), 10% okara–SPI gels; CO, control SPI gel (14.40% SPI); UO, WG-untreated okara–SPI gel (14.40% SPI and 1.60% WG-untreated okara); WGO, WG-treated okara–SPI gel (14.40% SPI and 1.60% WG-treated okara). (**d**), 15% okara–SPI gels; CO, control SPI gel (13.60% SPI); UO, WG-untreated okara–SPI gel (13.60% SPI and 2.40% WG-untreated okara); WGO, WG-treated okara–SPI gel (13.60% SPI and 2.40% WG-treated okara). Different letters indicate significant differences (*p* < 0.05).

**Figure 2:**
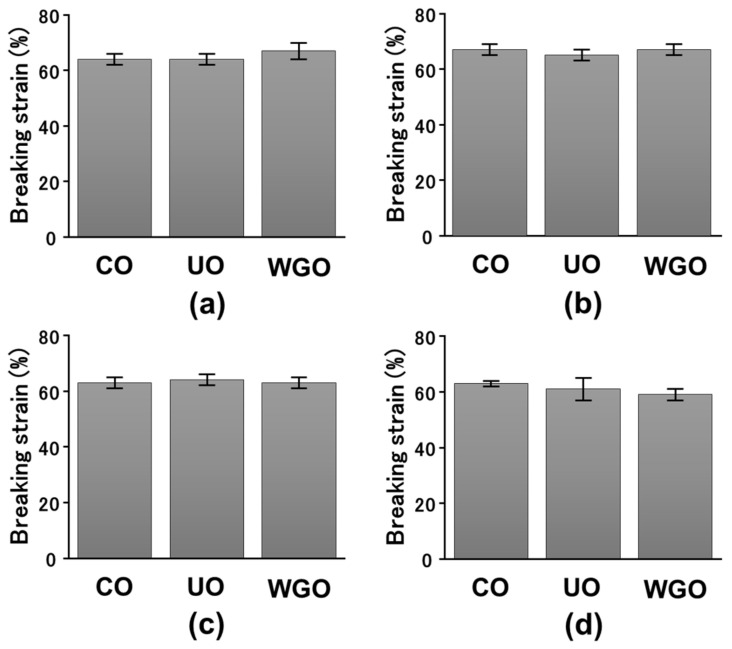
Breaking strain in okara–soybean protein isolate (SPI) gels. (**a**), 3% okara–SPI gels; (**b**), 5% okara–SPI gels; (**c**), 10% okara–SPI gels; (**d**), 15% okara–SPI gels. CO, control SPI gel; UO, wet-type grinder (WG)-untreated okara–SPI gel; WGO, WG-treated okara–SPI gel. SPI concentrations and WG-untreated and treated okara in 3%, 5%, 10%, and 15% okara–SPI gels are indicated in the Figure 1 legend.

Heat-induced SPI gels were prepared by adding 3%, 5%, 10%, and 15% WG-untreated or treated low-protein okara, and their mechanical properties were measured using compression measurements (Figure 1 and Figure 2). The breaking stress of the SPI gels after adding WG-untreated and treated okara increased, whereas the breaking strain of these gels did not increase. The breaking stress of the SPI gels containing WG-treated okara was higher than that of the SPI gels containing WG-untreated okara. These results indicate that WG- treated okara increases the breaking stress of the SPI gels but not the breaking strain. In our previous study, we demonstrated that the addition of WG-treated okara increases the breaking stress and strain of magnesium chloride-induced SPI gels. This effect increased with the increase in number of WG treatments. The breaking stress and strain of WG-treated okara–SPI gels also increased with the increase in concentration of WG-treated okara [12]. In addition, the effect of CNC and microcrystalline cellulose (MCC) on the gel properties of glucono-δ-lactone-induced SPI gels was investigated. The gel strength and storage modulus *G′* of CNC-SPI gels increased with the increase in CNC concentration from 0% to 0.75%, whereas the gel strength and storage modulus *G′* of MCC-SPI gels decreased [15]. The results in the present study are in line with these studies.

### 2.2. Effect of WG-Treated Okara on the Intermolecular Forces of SPI Gels

**Figure 3:**
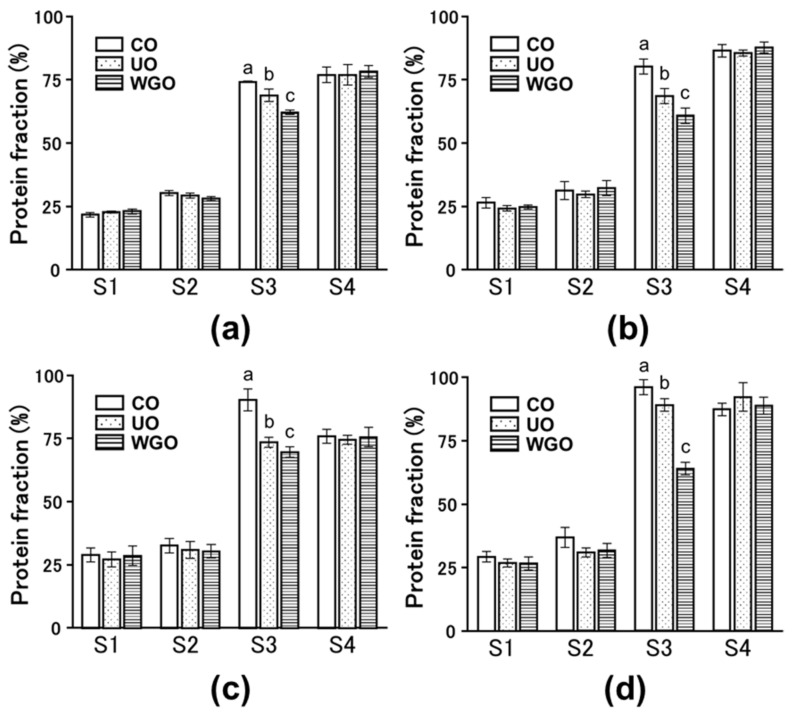
The protein solubility by homogenizing the okara–soybean protein isolate (SPI) gels in the four solutions (S1–S4). (**a**), 3% okara–SPI gels; (**b**), 5% okara–SPI gels; (**c**), 10% okara–SPI gels; (**d**), 15% okara–SPI gels. S1, 0.6 M sodium chloride (NaCl); S2, 0.6 M NaCl and 1.5 M urea; S3, 0.6 M NaCl and 8.0 M urea; S4, 1.0 M sodium hydroxide. CO, control SPI gel; UO, wet grinder (WG)-untreated okara–SPI gel; WGO, WG-treated okara–SPI gel. SPI concentrations and WG-untreated and treated okara in 3%, 5%, 10%, and 15% okara–SPI gels are indicated in the Figure 1 legend. Different letters indicate significant differences (*p* < 0.05).

Protein solubility in salts, denaturants, and reducing agents was investigated to understand molecular forces involved in the formation of protein gels [16]. Deng et al. [17] assessed protein solubility by homogenizing egg white gels in four different solutions (S1, 0.6 M NaCl; S2, 0.6 M NaCl and 1.5 M urea; S3, 0.6 M NaCl and 8.0 M urea; and S4, 0.6 M NaCl, 8.0 M urea, and 0.5 M 2-mercaptoethanol (2-ME)) to evaluate electrostatic interactions, hydrogen bonds, hydrophobic interactions, and disulfide bonds, respectively [17]. In this study, we used 1.0 M NaOH as S4 and examined protein solubility by homogenizing SPI gels in four different solutions. There were no differences in protein solubility in S1, S2, and S4 among the untreated, WG-untreated okara, and WG-treated okara–SPI gels. However, the protein solubility of WG-treated okara–SPI gels in S3 was lower than that of the without okara and with WG-untreated okara added SPI gels (Figure 3). 

The two major components of SPI are 7S globulin (7S) and 11S globulin (11S). The effects of various reagents on the formation of the heat-induced gels of 7S, 11S, and SPI have been previously studied [18,19,20]. Electrostatic interactions and disulfide bonds are involved in the formation of 11S gels, whereas hydrogen bonding and hydrophobic interactions primarily contribute to the formation of SPI gels [18]. In the 7S gelation process, gel formation was inhibited during the heating process with increasing concentrations of sodium thiocyanate, which hindered hydrophobic interactions. Furthermore, the gel was not formed in the presence of more than 4 M guanidine hydrochloride, which prevented hydrogen bonding and hydrophobic interactions. These results suggest that hydrogen bonding and hydrophobic interactions are involved in the formation of 7S gels [19]. The role of disulfide bonding in the mechanical properties and gel structures of 11S and SPI was also studied using 2-ME, which inhibited disulfide bond formation. The 11S gels showed a higher storage modulus *G′* and denser gel networks than those of the SPI gels. With the increase in concentration of 2-ME, the storage modulus *G′* and density of the gel structures of 11S gels decreased, but those of the SPI gels did not change significantly [20]. These studies indicated that hydrogen bonding and hydrophobic interactions are primarily involved in forming SPI gels. 

In this study, the WG-treated okara strengthened the intermolecular forces involved in the formation SPI gels that were solubilized in S3 (0.6 M NaCl and 8 M urea). In contrast, there are no differences in protein solubility by S2 (0.6 M NaCl and 1.5 M urea) (Figure 3). According to Pérez-Mateos et al. [16], 1.5 M urea reduced hydrogen bonding, whereas 8.0 M urea decreased hydrogen bonding and hydrophobic interactions. It is suggested that during the formation of SPI gels, hydrophobic interactions are enhanced by the addition of WG-treated okara [16,17].

### 2.3. Relationship between Mechanical Properties and Intermolecular Forces in Okara–SPI Gels

**Figure 4:**
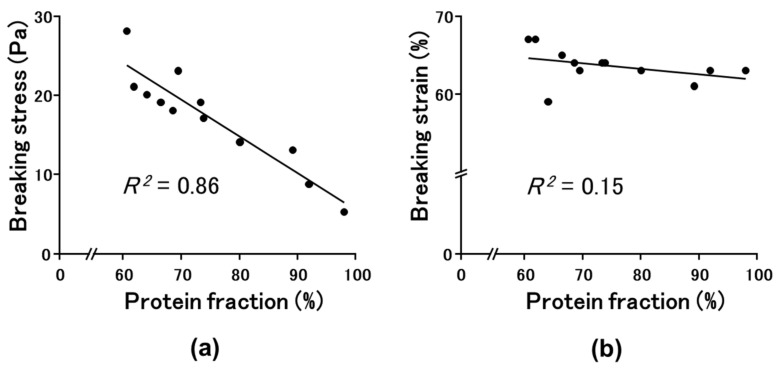
The relationship between breaking stress (**a**) or strain (**b**) and protein solubility in S3 (0.6 M NaCl and 8.0 M urea).

Because upon addition of WG-treated okara the breaking stress of the SPI gel increased, and the protein solubility of the SPI gel in S3 decreased compared with those of the SPI gels without okara or with WG-untreated okara, we examined the relationship between breaking stress or strain and protein solubility in S3 (Figure 4). A negative correlation (*R*^2^ = 0.86) was observed between breaking stress and protein solubility in S3, whereas no correlation was observed between breaking strain and protein solubility in S3. 

In previous studies, Fourier transform infrared analysis revealed that the band at 1618 cm^−1^ (associated with the intermolecular β-sheet structure) increased with the formation of 7S and 11S heat-induced gels. In 7S and 11S, the value of the storage modulus *G′* correlated well with the increase in the band at 1618 cm^−1^ (*R* = 0.93) [21]. Additionally, the effect of sugarcane fiber, wheat bran cellulose, or CNC on the rheological properties and structure of SPI or myofibrillar protein gels was investigated. Adding these fibers to protein gels increased their water-holding capacity and gel strength. Raman spectra showed that the formation of the β-sheet structure increased with increasing concentrations of these fibers [22,23,24]. These studies suggest that WG-treated okara enhances the intermolecular β-sheet structure formed in the WG-treated okara–SPI gels. 

## 3. Conclusions

SPI gels were prepared with varying amounts of WG-treated okara, and compression measurements were performed. WG-treated okara increased the breaking stress but not the breaking strain of SPI gels. Protein solubility was examined by homogenizing the gels in four different solutions. WG-treated okara strengthened the intermolecular forces that were homogenized in S3 (0.6 M NaCl and 8.0 M urea) to form SPI gels. A significant negative correlation was observed between breaking stress and protein solubility in S3 (*R*^2^ = 0.86). These results suggest that the WG-treatd okara increases breaking stress and enhances the hydrophobic interactions of SPI gels. This study will pave the way for future studies on developing protein-gel-based food using nano-fiber technologies.

## 4. Materials and Methods

### 4.1. Materials

Defatted okara (Newproplus 1000) and SPI (Fujipro F) were obtained from Fuji Oil (Izumisano, Japan). According to the manufacturer, Newproplus 1000 contains 6.0% water, 20.7% protein, 0.2% fat, 69.1% carbohydrates (63.7% dietary fiber), and 4.0% ash; Fujipro F contains 5.0% water, 86.3% protein, 0.2% fat, 4.0% carbohydrates, and 4.5% ash.

### 4.2. Preparation of Low-Protein Okara and WG-Treated Okara

**Figure 5:**
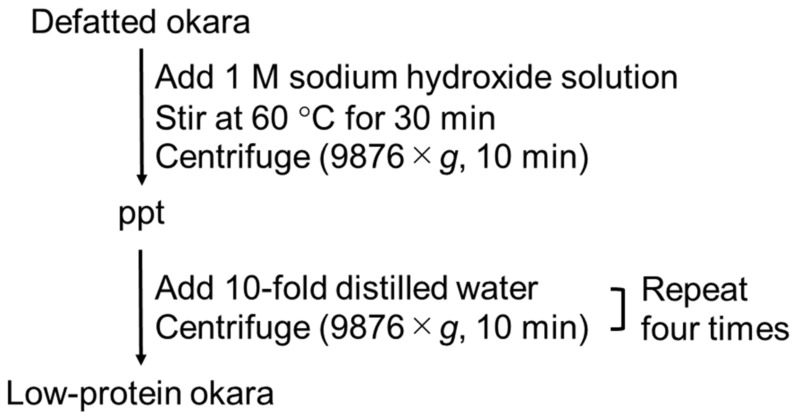
Schematic diagram for the preparation procedure of low-protein okara.

The schematic diagram of the preparation procedure for low-protein okara is shown in Figure 5. A 1 M sodium hydroxide (NaOH) solution was added to the okara and stirred at 60 °C for 30 min to elute the proteins. The alkali-treated okara was centrifuged (9876× *g*, 10 min) to obtain the precipitate. Next, the precipitate was centrifuged four times with 10-fold distilled water. The amount of protein was determined using the Kjeldahl method and was found to be reduced from 17.54 ± 0.72% to 2.38 ± 0.01%. For WG-treated okara preparation, the 2 wt% low-protein okara dispersion was pulverized five times using a WG (MKCA6-2, Masuko Sangyo, Kawaguchi, Japan) with a − 0.15 mm gap at 1540 rpm of the stone disk.

### 4.3. Preparation of Okara–SPI Gels

**Figure 6:**
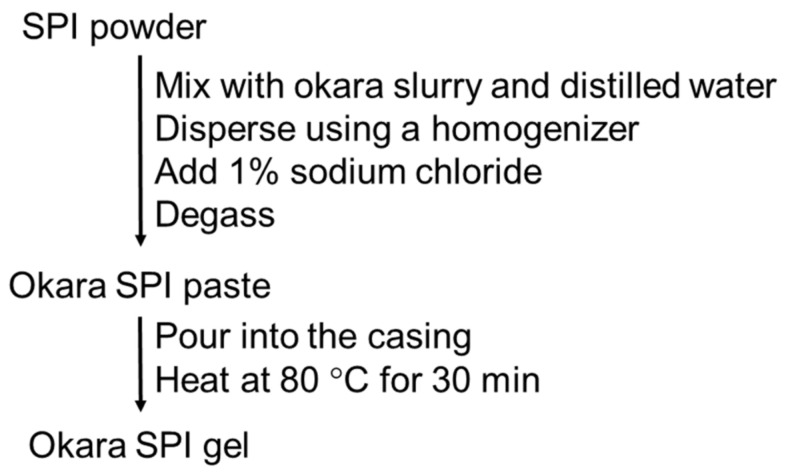
Schematic diagram of the preparation procedure for okara-soybean protein isolate (SPI) gels.

The preparation procedure for okara-SPI gels is shown in Figure 6. The total content of okara and SPI was adjusted to 16% and okara and concentrations of WG-treated or unteated okara and SPI for the preparation of okara–SPI gels are shown in Table 1. For okara–SPI gel preparation, SPI powder was hydrated by mixing with the WG-treated okara slurry and dispersed using a homogenizer (IKA Ultra-Turrax T8 Disperser, IKA Japan K.K., Higashiosaka, Japan). After adding 1% sodium chloride (NaCl), the sol was degassed and poured into the casing (inner diameter: 30 mm). The casing was heated at 80 °C for 30 min in a water bath (FSGPD05; Fisher Scientific International, Hampton, NH, USA).

### 4.4. Compression Measurements

Compression measurements were performed using a texture analyzer (TA-XT2iHR, Stable Micro Systems, Godalming, Surrey, UK) attached to a 5 kg load cell at 25 °C. A cylindrical plunger with a diameter of 50 mm was used to sample the gels (30 mm diameter and 30 mm height) at a compression speed of 1 mm/s. At least six gels were examined in each experiment.

### 4.5. Protein Solubility

To evaluate the intermolecular forces involved in the formation of a gel, the protein solubility in four different solutions (S1, S2, S3, and S4) was examined using a protocol modified from a method described by Deng et al. [17]; S1, 0.6 M NaCl; S2, 0.6 M NaCl and 1.5 M urea; S3, 0.6 M NaCl and 8.0 M urea; and S4, 1.0 M NaOH [16]. 

Gels (1 g) in a 9 mL solution (S1, S2, S3, or S4) were homogenized at 12,000 rpm for 30 s and centrifuged (20,000× *g*, 10 min). The amount of protein in the supernatant was determined using the BCA protein assay kit (Thermo Fisher Scientific, Waltham, MA, USA). At least three gels were examined at each point in each experiment.

### 4.6. Statistical Analyses

Data represent the mean ± standard deviation. Statistical significance was calculated using one-way analysis of variance followed by Tukey’s post hoc test using the Origin 2020b software (Origin Lab, Northampton, MA, USA). The data were considered statistically significant at *p* < 0.05.

## Figures and Tables

**Table 1:** Concentrations of wet-type grinder (WG)-treated or untreated okara and soybean protein isolate (SPI) for the preparation of okara–SPI gels.

	WG-Treated or Untreated Okara	SPI
3% okara–SPI	0.48%	15.52%
5% okara–SPI	0.80%	15.20%
10% okara–SPI	1.60%	14.40%
15% okara–SPI	2.40%	13.60%

The total content of okara and SPI was adjusted to 16%.

## Data Availability

Not applicable.
